# (p)ppGpp and CodY Promote Enterococcus faecalis Virulence in a Murine Model of Catheter-Associated Urinary Tract Infection

**DOI:** 10.1128/mSphere.00392-19

**Published:** 2019-07-24

**Authors:** C. Colomer-Winter, A. L. Flores-Mireles, S. Kundra, S. J. Hultgren, J. A. Lemos

**Affiliations:** a Department of Oral Biology, University of Florida College of Dentistry, Gainesville, Florida, USA; b Department of Molecular Microbiology, Washington University School of Medicine, St. Louis, Missouri, USA; c Center for Women’s Infectious Disease Research, Washington University School of Medicine, St. Louis, Missouri, USA; University of Rochester

**Keywords:** (p)ppGpp, CAUTI, CodY, *Enterococcus*, stringent response

## Abstract

In *Firmicutes*, the nutrient-sensing regulators (p)ppGpp, the effector molecule of the stringent response, and CodY work in tandem to maintain bacterial fitness during infection. Here, we tested (p)ppGpp and *codY* mutant strains of Enterococcus faecalis in a catheter-associated urinary tract infection (CAUTI) mouse model and used global transcriptional analysis to investigate the relationship of (p)ppGpp and CodY. The absence of (p)ppGpp or single inactivation of *codY* led to lower bacterial loads in catheterized bladders and diminished biofilm formation on fibrinogen-coated surfaces under *in vitro* and *in vivo* conditions. Single inactivation of the bifunctional (p)ppGpp synthetase/hydrolase *rel* did not affect virulence, supporting previous evidence that the association of (p)ppGpp with enterococcal virulence is not dependent on the activation of the stringent response. Inactivation of *codY* in the (p)ppGpp^0^ strain restored E. faecalis virulence in the CAUTI model as well as the ability to form biofilms *in vitro*. Transcriptome analysis revealed that inactivation of *codY* restores, for the most part, the dysregulated metabolism of (p)ppGpp^0^ cells. While a clear linkage between (p)ppGpp and CodY with expression of virulence factors could not be established, targeted transcriptional analysis indicates that a possible association between (p)ppGpp and c-di-AMP signaling pathways in response to the conditions found in the bladder may play a role in enterococcal CAUTI. Collectively, data from this study identify the (p)ppGpp-CodY network as an important contributor to enterococcal virulence in catheterized mouse bladder and support that basal (p)ppGpp pools and CodY promote virulence through maintenance of a balanced metabolism under adverse conditions.

**IMPORTANCE** Catheter-associated urinary tract infections (CAUTIs) are one of the most frequent types of infection found in the hospital setting that can develop into serious and potentially fatal bloodstream infections. One of the infectious agents that frequently causes complicated CAUTI is the bacterium Enterococcus faecalis, a leading cause of hospital-acquired infections that are often difficult to treat due to the exceptional multidrug resistance of some isolates. Understanding the mechanisms by which E. faecalis causes CAUTI will aid in the discovery of new druggable targets to treat these infections. In this study, we report the importance of two nutrient-sensing bacterial regulators, named (p)ppGpp and CodY, for the ability of E. faecalis to infect the catheterized bladder of mice.

## Abstract (precis)

Catheter-associated urinary tract infections (CAUTIs) are one of the most frequent types of infection found in the hospital setting that can develop into serious and potentially fatal bloodstream infections. One of the infectious agents that frequently causes complicated CAUTI is the bacterium Enterococcus faecalis, a leading cause of hospital-acquired infections that are often difficult to treat due to the exceptional multidrug resistance of some isolates. Understanding the mechanisms by which E. faecalis causes CAUTI will aid in the discovery of new druggable targets to treat these infections. In this study, we report the importance of two nutrient-sensing bacterial regulators, named (p)ppGpp and CodY, for the ability of E. faecalis to infect the catheterized bladder of mice.

## INTRODUCTION

Catheter-associated urinary tract infections (CAUTIs) are one of the most common hospital-acquired infections, accounting worldwide for about 40% of all nosocomial infections (1–3). In addition to substantially increasing hospitalization time and costs, CAUTI can lead to serious and potentially deadly secondary bloodstream infections (4). Complicated CAUTI is often the result of bacteria forming biofilms on indwelling urinary catheters, and enterococci (mainly Enterococcus faecalis and Enterococcus faecium) appear as the second leading cause of complicated CAUTI in many health care facilities (4–6). In addition, E. faecalis and E. faecium are major etiological agents of other life-threatening infections, such as infective endocarditis, and an even more serious threat to public health due to their exceptional antibiotic resistance (7). The recent rise in the number of enterococcal infections urges the development of new therapies, and understanding the mechanisms that promote E. faecalis CAUTI might uncover new druggable targets.

The pathogenic potential of E. faecalis, and of all enterococci in general, is tightly associated with its outstanding ability to survive an array of physical and chemical stresses, including common detergents and antiseptics; fluctuations in temperature, pH, and humidity; and prolonged starvation (7). The regulatory second messengers ppGpp (guanosine tetraphosphate) and pppGpp (guanosine pentaphosphate), collectively known as (p)ppGpp, broadly promote bacterial stress tolerance and virulence (8, 9). In E. faecalis, (p)ppGpp has been shown to promote virulence in invertebrate and vertebrate animal models and to mediate the expression of virulence-related traits such as growth in blood and serum, biofilm formation, intraphagocytic survival, and antibiotic tolerance (10–17). Originally described as the mediator of the stringent response (SR) (8), (p)ppGpp has distinct effects on bacterial physiology: at low (basal) concentrations, it fine-tunes bacterial metabolism to adjust cellular growth in response to mild environmental changes (18), whereas at high levels, it activates the SR responsible for promoting cell survival by slowing down growth-associated pathways and activating stress survival pathways (8, 18).

Two enzymes, the bifunctional synthetase/hydrolase Rel and the small alarmone synthetase RelQ, are responsible for enterococcal (p)ppGpp turnover (10, 19). Despite both Δ*rel* and Δ*rel* Δ*relQ* strains being unable to mount the SR (10, 13), there are fundamental differences in basal (p)ppGpp levels between these two strains. Specifically, while the double mutant, here called the (p)ppGpp^0^ strain, is completely unable to synthesize (p)ppGpp, basal (p)ppGpp levels are about 4-fold higher in the Δ*rel* strain due to the constitutive and weak synthetase activity of RelQ (10, 14, 19). Accumulated evidence indicates that the metabolic control exerted by basal (p)ppGpp pools is more important during enterococcal infections than the semidormancy state characteristic of the SR (10, 13–17). This is exemplified by the distinct virulence phenotypes of Δ*rel* and (p)ppGpp^0^ strains, both of which are unable to mount the SR. Specifically, while only the (p)ppGpp^0^ strain displayed attenuated virulence in Caenorhabditis elegans (10), Galleria mellonella (11, 13, 16), and a rabbit abscess model (15), the Δ*rel* single mutant strain showed attenuated virulence in a rabbit model of infective endocarditis (17).

In low-GC Gram-positive bacteria such as E. faecalis, (p)ppGpp controls the transcription of nutrient uptake and amino acid biosynthesis genes via the branched-chain-amino-acid (BCAA)- and GTP-sensing CodY regulator (20). It follows that (p)ppGpp accumulation during BCAA starvation severely depletes intracellular GTP pools in all *Firmicutes* such that CodY regulation is severely impaired due to the depletion of its two cofactors (20). We recently confirmed the existence of the (p)ppGpp-CodY network in E. faecalis and demonstrated that inactivation of *codY* restored several phenotypes of the (p)ppGpp^0^ mutant strain, including virulence in G. mellonella (16). However, the contribution of the global nutritional regulator CodY to enterococcal pathogenesis in mammalian hosts remains unknown.

In this work, we examined the contribution of (p)ppGpp and CodY to the pathogenesis of E. faecalis in a murine CAUTI model (21). We discovered that, separately, basal levels of (p)ppGpp and the transcriptional regulator CodY promote biofilm formation in urine under *in vitro* conditions as well as virulence in a murine CAUTI model. Global transcriptome analysis validates previous findings that deletion of *codY* restores, at least in part, the dysregulated metabolism of the cell in the absence of (p)ppGpp. Finally, targeted mRNA quantifications reveal that the (p)ppGpp-CodY network alters the expression of genes coding for cyclic di-AMP (c-di-AMP) biosynthetic enzymes and of CAUTI virulence factors. Altogether, data from this study identify the (p)ppGpp-CodY network as a contributor to enterococcal catheter colonization in the urinary tract and further support that basal levels of (p)ppGpp promote bacterial virulence through maintenance of a balanced bacterial metabolism.

## RESULTS

### The (p)ppGpp^0^ and Δ*codY* strains show impaired colonization in a murine CAUTI model.

**FIG 1:**
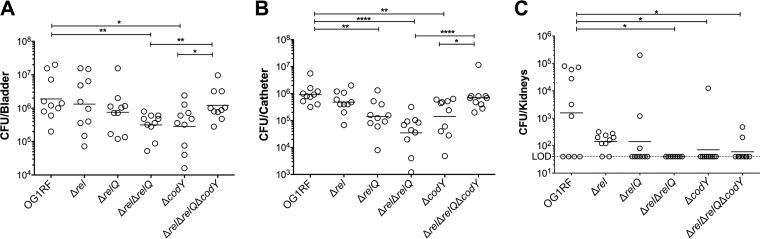
(p)ppGpp and CodY promote virulence of E. faecalis in a murine CAUTI model. The parent strain OG1RF and its derivatives were inoculated into the bladders of mice immediately after catheter implantation (*n* = 10). After 72 h, animals were euthanized, and bacterial burdens in bladders (A), catheters (B), and kidneys (C) were quantified. Graphs show total CFU recovered from these sites, each symbol represents an individual mouse, and the median value is shown as a horizontal line. Symbols on the dashed line indicate that recovery was below the limit of detection (LOD) (40 CFU). The data were pooled from two independent experiments. Two-tailed Mann-Whitney U tests were performed to determine significance (*, *P < *0.05; **, *P < *0.005; ****, *P < *0.0001).

We compared the abilities of the E. faecalis OG1RF parent, Δ*rel*, Δ*relQ*, (p)ppGpp^0^, Δ*codY*, and (p)ppGpp^0^ Δ*codY* strains to colonize and persist in the bladder of catheterized mice. Briefly, catheters were implanted into the bladders of C57BL/6Ncr mice prior to transurethral inoculation with ∼2 × 10^7^ CFU of each designated strain. At 3 days postinfection, the OG1RF strain was readily recovered from bladders (6.3 ± 0.6 log_10_ CFU) and catheters (6.0 ± 0.3 log_10_ CFU) of all infected animals (Fig. 1). Inactivation of *rel* (Δ*rel*) or *relQ* (Δ*relQ*) did not significantly alter E. faecalis colonization in bladders, but the level of recovery of Δ*relQ* bacteria from catheters was significantly lower (∼0.8-log_10_ reduction; *P* = 0.0027 compared to OG1RF). On the other hand, the (p)ppGpp^0^ strain displayed ∼0.8-log_10_ (*P* = 0.0015) and 1.5-log_10_ (*P < *0.0001) reductions in CFU recovered from bladders and catheters, respectively (Fig. 1). The Δ*codY* strain phenocopied the (p)ppGpp^0^ strain, showing ∼0.8-log_10_ reductions in CFU recovered from both bladders (*P = *0.0156) and catheters (*P = *0.0031) (Fig. 1). However, inactivation of *codY* in the (p)ppGpp^0^ background [(p)ppGpp^0^ Δ*codY* triple mutant] restored bacterial bladder and catheter colonization to near-parent-strain levels. Despite great variations from animal to animal but in relative agreement with the bacterial loads detected on catheters and in bladders, the OG1RF and Δ*rel* strains were consistently able to ascend to the kidneys (Fig. 1C). However, the (p)ppGpp^0^ Δ*codY* triple mutant failed to consistently ascend to the kidneys, suggesting that the virulence of this strain may be partially compromised.

### (p)ppGpp promotes timely growth of E. faecalis in human urine.

**FIG 2:**
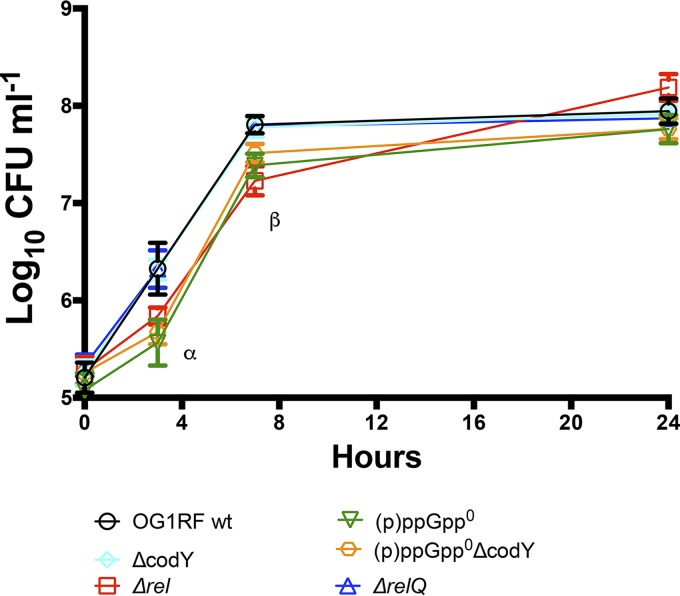
(p)ppGpp supports timely growth of E. faecalis in human urine. Growth of the parent E. faecalis strain OG1RF and its derivative mutant strains in pooled human urine was analyzed. Aliquots at selected time points were serially diluted and plated onto TSA plates for CFU enumeration. The graph shows the average log_10_-transformed CFU (means and standard deviations) from three independent experiments. Mutant strains were compared to wild-type (wt) strain OG1RF by two-way ANOVA with Dunnett’s multiple-comparison test. Asterisks indicate significant differences at 3 h of incubation for the Δ*rel*, (p)ppGpp^0^, and (p)ppGpp^0^ Δ*codY* strains and at 7 h of incubation for the Δ*rel* and (p)ppGpp^0^ Δ*codY* strains (*P* < 0.0001).

The (p)ppGpp^0^ strain was previously shown to have growth and survival defects in whole blood and serum (15, 16). Interestingly, inactivation of *codY* in the (p)ppGpp^0^ background [(p)ppGpp^0^ Δ*codY*] restored the (p)ppGpp^0^ growth defect in blood but not in serum (16). Here, we tested the ability of (p)ppGpp-defective [Δ*rel*, Δ*relQ*, and (p)ppGpp^0^] and *codY* [Δ*codY* and (p)ppGpp^0^ Δ*codY*] strains to replicate in pooled human urine *ex vivo*. Despite the colonization defect in the murine CAUTI model (Fig. 1), the Δ*codY* strain grew as well as the parent and Δ*relQ* strains (Fig. 2). On the other hand, Δ*rel*, (p)ppGpp^0^, and (p)ppGpp^0^ Δ*codY* strains grew slower in the first 12 h of incubation, with an ∼0.6-log_10_ CFU difference at 3 and 7 h postinoculation (Fig. 2). Nevertheless, upon entering stationary phase, all strains reached similar growth yields and remained viable for at least 24 h.

### (p)ppGpp and CodY support biofilm formation in urine.

**FIG 3:**
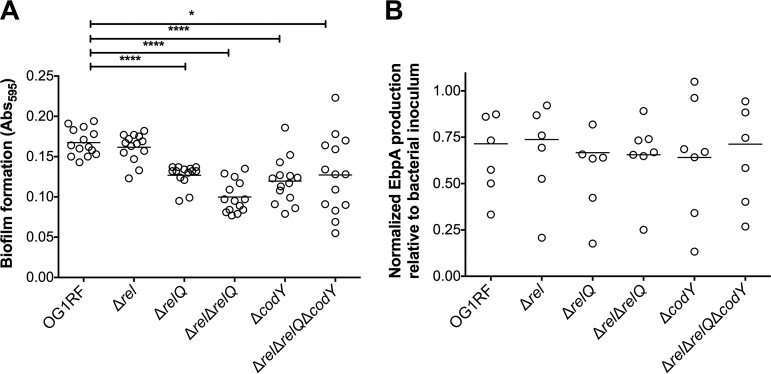
The (p)ppGpp-CodY network contributes to biofilm formation in urine. (A) Fibrinogen-coated 96-well polystyrene plates were incubated with E. faecalis strains for 48 h in human urine supplemented with 20 mg ml^−1^ BSA. Plates were stained with 0.5% crystal violet, which was subsequently dissolved with 33% acetic acid, and the absorbance at 595 nm was measured. (B) EbpA quantification in urine. Strains were grown in urine plus BSA overnight prior to quantification of EbpA by an ELISA. EbpA surface exposure on E. faecalis cells was detected using mouse anti-EbpA^Full^ and HRP-conjugated goat anti-rabbit antisera, and the absorbance was determined at 450 nm. EbpA production titers were normalized against the bacterial titers. Experiments were performed independently in triplicate and analyzed by a two-tailed Mann-Whitney U test (*, *P < *0.05; ****, *P < *0.0001).

Biofilm formation on urinary catheters is critical for enterococcal CAUTI (22–24). This is exemplified by the observation that E. faecalis OG1RF requires the presence of a catheter to persist for more than 48 h in murine bladders (21). Follow-up studies revealed that catheterization, in mice and in humans, triggers an inflammatory response that releases the host protein fibrinogen, which is used by E. faecalis as a nutrient as well as a scaffold to adhere to and colonize the catheter surface (22–25). Taking into account that the reduction in bacterial counts of Δ*relQ*, (p)ppGpp^0^, and Δ*codY* strains was more pronounced on catheters than in bladders (Fig. 1) and that (p)ppGpp supports long-term survival of E. faecalis in biofilms (12), we sought to investigate if the attenuated virulence of these strains in murine CAUTI was linked to a reduced ability to form biofilms on fibrinogen-coated surfaces in the presence of urine. Since catheterization normally elicits proteinuria in the host, and E. faecalis requires a protein source to optimally form biofilms in urine (22, 24), human urine was supplemented with bovine serum albumin (BSA). In agreement with the bacterial loads obtained from the implanted catheters (Fig. 1B), quantifications of E. faecalis biofilm biomass revealed that Δ*relQ*, (p)ppGpp^0^, and Δ*codY* strains were deficient in biofilm production, while the Δ*rel* strain phenocopied the parent OG1RF strain (Fig. 3A). Notably, inactivation of *codY* in the (p)ppGpp^0^ background alleviated the defective phenotype of the (p)ppGpp^0^ strain, albeit the differences between the (p)ppGpp^0^ Δ*codY* and OG1RF strains were still statistically significant.

As indicated above, the main factor involved in E. faecalis biofilm formation on urinary catheters is the production of the Ebp pilus, which binds directly to fibrinogen via the EbpA N-terminal domain (22, 26, 27). Quantification of EbpA via an enzyme-linked immunosorbent assay (ELISA) showed no noticeable reductions in EbpA production in any of the mutant strains (Fig. 3B), suggesting that the (p)ppGpp-CodY network promotes biofilm formation of fibrinogen-coated surfaces in an Ebp-independent manner.

### Inactivation of *codY* restores the dysregulated metabolism of the (p)ppGpp^0^ strain.

Previously, we proposed that the virulence attenuation of the (p)ppGpp^0^ strain in different animal models is, in large part, due to the dysregulated metabolism caused by a lack of (p)ppGpp control (13, 14, 16). By monitoring H_2_O_2_ production and the culture pH, we provided the first evidence that inactivation of *codY* restored a balanced metabolism to the (p)ppGpp^0^ background strain (16). To obtain additional insights into the (p)ppGpp-CodY relationship, we used RNA sequencing (RNA-seq) technology to compare the transcriptomes of OG1RF, (p)ppGpp^0^, and (p)ppGpp^0^ Δ*codY* strains grown to mid-exponential phase in chemically defined FMC medium (28) supplemented with 10 mM glucose (FMCG medium). In the (p)ppGpp^0^ strain, 690 genes were differentially expressed compared to OG1RF (*P < *0.05; 2-fold cutoff) (see Table S1 in the supplemental material), representing ∼27% of the entire E. faecalis OG1RF genome. In agreement with a previous microarray analysis (14), multiple phosphotransferase systems (PTSs) as well as citrate, glycerol, malate, and serine utilization pathways were strongly induced in the (p)ppGpp^0^ strain under these conditions. A selected and representative number of dysregulated transport and metabolic genes in the (p)ppGpp^0^ strain are shown in Tables 1 and 2, respectively. The upregulation of alternate carbon metabolism genes that are expected to be under carbon catabolite repression (CCR) under the glucose-rich conditions of FMCG medium indicates that a complete lack of (p)ppGpp places E. faecalis in what we have originally termed a “transcriptionally relaxed” state (14). Compared to the parent strain, 737 genes (∼29% of the entire genome) were differentially expressed in the (p)ppGpp^0^ Δ*codY* triple mutant strain (*P < *0.05; 2-fold cutoff) (Table S2). Despite the even larger number of differentially expressed genes, inactivation of *codY* in the (p)ppGpp^0^ background strain normalized the transcription of 274 genes that were dysregulated in the (p)ppGpp^0^ background (Table S3). The great majority (∼95%) of these genes were upregulated in the (p)ppGpp^0^ strain, supporting that deletion of *codY* abrogates, at least in part, the transcriptionally relaxed state of the (p)ppGpp^0^ mutant. More specifically, inactivation of *codY* in the (p)ppGpp^0^ background normalized the transcription (or brought it to levels much closer to that of the parent strain) of ∼70 transport systems as well as over 130 metabolic genes, including citrate, glycerol, malate, and serine utilization pathways; dehydrogenases; and molybdenum-dependent enzymes (Table S3).

**TABLE 1:** Transcriptional restoration of selected nutrient transport systems upon inactivation of *codY* in the (p)ppGpp^0^ background

Locus or gene name	Function(s)	Fold change^*a*^	
		(p)ppGpp^0^	(p)ppGpp^0^ Δ*codY*
PTSs			
OG1RF_10341	PTS	+5.5	
OG1RF_10346	PTS	+4.8	
OG1RF_10433	PTS	+5.1	
OG1RF_10746	PTS	+8.2	
OG1RF_11236	PTS	+3.3	
OG1RF_11249	PTS	+3.1	−3.3
OG1RF_11512	PTS	+5.0	
OG1RF_11614	PTS	+4.2	
OG1RF_11780	PTS	+3.8	
OG1RF_12261	PTS	+4.6	
*bglP*	PTS, β-glucoside uptake	+7.4	
*frwB*	PTS, fructose uptake	+11.5	
*malX*	PTS, maltose uptake	+4.7	
*mltF2*	PTS, mannitol uptake	+7.1	
*scrA*	PTS, β-glucoside uptake	+3.4	
*sorA*	PTS, sorbose uptake	+32.5	
*treB*	PTS, trehalose uptake	+11.9	−2.0
*ulaB*	PTS, ascorbate uptake	+5.1	

ABC-type transporters			
OG1RF_10665	ABC-type transporter	+3.4	−2.5
OG1RF_10879	ABC-type transporter	+2.2	
OG1RF_11003	ABC-type transporter	+15.9	
OG1RF_11188	ABC-type transporter	+8.1	
OG1RF_11763	ABC-type transporter	+3.2	
OG1RF_11774	Sugar ABC-type transporter	+38.5	
OG1RF_12466	ABC-type transporter	+3.5	
*mdlB*	Multidrug ABC-type transporter	+3.2	
*modB*	Molybdenum transporter	+31.5	
T0	Oligopeptide transporter	−3.3	
*ziaA*	Zinc ABC-type transporter	+3.2	

Other porters			
OG1RF_11781	Sodium symporter	+5.3	
OG1RF_11873	Phosphate transporter	+4.5	−2.5
OG1RF_11954	Xanthine/uracil permease	+3.0	
OG1RF_12019	Gluconate symporter	+4.7	
OG1RF_12274	Major facilitator transporter	+21.3	
OG1RF_12280	Cytosine/purine permease	+5.4	
OG1RF_12572	Citrate transporter	+51.8	
*dctM*	Organic acid transporter	+4.5	
*glpF2*	Glycerol uptake	+11.0	

a Values represent fold changes in transcription compared to the parent strain. All values shown were statistically significant (*P *<* *0.05). Blank fields indicate that there were no significant differences between mutant and parent strains.

**TABLE 2:** Transcriptional profile of selected metabolic genes upon inactivation of *codY* in the (p)ppGpp^0^ background

Locus or gene name	Function	Fold change^*a*^	
		(p)ppGpp^0^	(p)ppGpp^0^ Δ*codY*
Citrate metabolism			
OG1RF_10979	Citrate carrier	+31.2	
*citC*	Citrate metabolism	+17.0	−5.0
*citD*	Citrate metabolism	+18.7	−3.3
*citE*	Citrate metabolism	+15.6	−5.0
*citF*	Citrate metabolism	+15.0	−5.0
*citX*	Citrate metabolism	+13.4	−5.0
*citXG*	Citrate metabolism	+13.2	−2.0

Serine metabolism			
*sdaA*	Serine dehydratase	+103.5	
*sdaB*	Serine dehydratase	+94.5	−2.5

Glycerol metabolism			
*glpK*	Glycerol kinase	+5.7	−2.5
*glpO*	Glycerol-3-phosphate oxidase	+6.6	−2.5
*gldA2*	Glycerol dehydrogenase	+13.7	

Molybdenum metabolism			
OG1RF_11183	MOSC protein^*b*^	+30.1	
OG1RF_11185	Molybdopterin-binding protein	+25.6	
OG1RF_11944	Molybdenum hydroxylase	+29.7	
OG1RF_11951	Molybdenum hydroxylase	+10.8	−2.0
*moaA*	Molybdenum cofactor synthesis	+20.0	
*moaB*	Molybdenum cofactor synthesis	+27.1	
*moaC*	Molybdenum cofactor synthesis	+10.1	
*yedF2*	Selenium metabolism	+23.3	
*ygfJ*	Molybdenum hydroxylase	+20.6	

Other metabolic genes			
OG1RF_11942	Ferredoxin NADP^+^ reductase	+23.4	
OG1RF_11943	Flavodoxin	+20.7	
*allD*	Ureidoglycolate dehydrogenase	+14.7	
*mdh*	Malate dehydrogenase	+16.5	
*fbp*	Fructose-1,6-bisphosphatase	+10.6	+3.9
*oadA*	Oxaloacetate decarboxylase	+14.2	−3.3
OG1RF_10107	Glycosyl hydrolase	+60.1	
*gcdB*	Glutaconyl-CoA decarboxylase	+15.0	−5.0
*arcC2*	Carbamate kinase	+17.4	
*gltA*	Glutamate synthase	+16.8	
OG1RF_11949	Cysteine desulfurase	+25.7	−2.5
*hydA*	Dihydropyrimidinase	+14.6	
OG1RF_11585	Ribosylpyrimidine nucleosidase	+11.1	−3.3
OG1RF_10108	Endoribonuclease	+48.7	
*endOF3*	*N*-Acetylglucosaminidase	+60.1	

a Values represent fold changes in transcription compared to the parent strain. All values shown were statistically significant (*P *<* *0.05). Blank fields indicate that there were no significant differences between mutant and parent strains.

b MOSC, MOCO (molybdenum cofactor) sulfurase C-terminal.

10.1128/mSphere.00392-19.1 TABLE S1 Genes differentially expressed in the (p)ppGpp^0^ strain in comparison to the parent OG1RF strain (*P ≤ *0.05; 2-fold cutoff). Download Table S1, XLSX file, 0.05 MB. Copyright © 2019 Colomer-Winter et al. 2019 Colomer-Winter et al. This content is distributed under the terms of the Creative Commons Attribution 4.0 International license.

10.1128/mSphere.00392-19.2 TABLE S2 Genes differentially expressed in the triple mutant Δ*codY* (p)ppGpp^0^ strain in comparison to the parent OG1RF strain (*P ≤ *0.05; 2-fold cutoff). Download Table S2, XLSX file, 0.05 MB. Copyright © 2019 Colomer-Winter et al. 2019 Colomer-Winter et al. This content is distributed under the terms of the Creative Commons Attribution 4.0 International license.

10.1128/mSphere.00392-19.3 TABLE S3 Side-by-side comparison of linear fold changes of selected genes in the (p)ppGpp^0^ strain and triple mutant Δ*codY* (p)ppGpp^0^ strain in comparison to the parent OG1RF strain. Download Table S3, XLSX file, 0.08 MB. Copyright © 2019 Colomer-Winter et al. 2019 Colomer-Winter et al. This content is distributed under the terms of the Creative Commons Attribution 4.0 International license.

### Dysregulation of c-di-AMP homeostasis may affect fitness of (p)ppGpp^0^ and Δ*codY* strains in urine.

**FIG 4:**
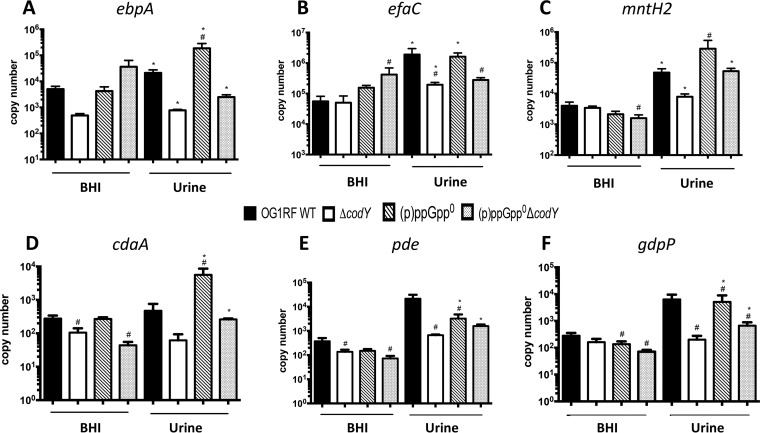
Transcript levels of selected genes in OG1RF, (p)ppGpp^0^, Δ*codY*, and (p)ppGpp^0^ Δ*codY* cultures in BHI broth or human urine. The E. faecalis OG1RF wild-type (WT), (p)ppGpp^0^, (p)ppGpp^0^ Δ*codY*, and Δ*codY* strains were grown in BHI broth to the mid-exponential growth phase. Cell pellets were washed thoroughly and then exposed to pooled human urine and fresh BHI broth for 30 min. The transcript levels of *ebpA* (A), *efaC* (B), *mntH2* (C), *cdaA* (D), *pde* (E), and *gdpP* (F) were determined by quantitative real-time PCR. The bar graphs show averages and standard deviations of results from three independent experiments performed in triplicate. Differences seen with the same strains under different conditions (#) or between parent and mutant strains under the same growth conditions (*) were compared via Student’s *t* test or ANOVA with a multiple-comparison test, respectively (*P* < 0.05).

Previously, the Ebp pilus and two high-affinity manganese transporters, EfaCBA and MntH2, were shown to be essential for E. faecalis virulence in a mouse CAUTI model (27, 29). In an attempt to identify (p)ppGpp- and CodY-dependent processes directly relevant to enterococcal CAUTI, we compared the transcriptional profiles of these virulence factors in parent and mutant strains. In brief, quantitative real-time PCR (qPCR) was used to compare expression levels of selected genes in exponentially grown brain heart infusion (BHI) broth cultures of OG1RF, Δ*codY*, (p)ppGpp^0^, and (p)ppGpp^0^ Δ*codY* strains before and after switching the cultures to pooled human urine. We found that the transcription of *ebpA* (coding for the pilin subunit), *efaC* (the ATP-binding subunit of the ABC-type transporter EfaCBA), and *mntH2* was strongly induced upon shifting the parent strain culture from BHI broth to urine (Fig. 4). While full upregulation of *efaC* in urine appears to be dependent on CodY, *ebpA* and *mntH2* transcription was differentially impacted by (p)ppGpp and CodY. Specifically, while inactivation of CodY (Δ*codY*) limited the induction of these genes after transition to urine, the loss of (p)ppGpp induced the transcription of *ebpA* and *mntH2* by ∼10-fold. The simultaneous inactivation of *codY* in the (p)ppGpp^0^ background [(p)ppGpp^0^ Δ*codY*] modestly raised *ebpA* but fully restored *mntH2* mRNA levels (Fig. 4).

We also assessed transcription levels of genes coding for enzymes involved in the metabolism of c-di-AMP, a nucleotide second messenger essential for osmotic stress survival and whose regulatory network appears to be intertwined with the (p)ppGpp regulatory network in other bacteria (30–34). In the OG1RF strain, transcription of the c-di-AMP cyclase *cdaA* was not significantly altered upon transition from BHI broth to urine; however, transcription of both c-di-AMP hydrolases (*pde* and *gdpP*) was induced by ∼50- and 100-fold, respectively (Fig. 4). While induction of *gdpP* was dependent on CodY, activation of *pde* in urine was not as robust in all mutant strains. Unexpectedly, *cdaA* transcription was strongly induced (∼50-fold) in the (p)ppGpp^0^ strain when shifted from BHI broth to urine, suggesting that c-di-AMP levels may be dysregulated in the (p)ppGpp^0^ strain during CAUTI. Notably, pools of c-di-AMP must be tightly controlled given that it is essential for growth but also highly toxic when present at high concentrations (35).

## DISCUSSION

In this report, we show that basal (p)ppGpp pools and the transcriptional regulator CodY mediate the virulence of E. faecalis in a murine CAUTI model. These results corroborate previous findings that changes in basal levels of (p)ppGpp contribute to the virulence of E. faecalis (10, 11, 13, 15, 17) and show, for the first time, that CodY regulation is also important for the virulence of E. faecalis. Despite the attenuated virulence of the Δ*codY*, Δ*relQ*, and (p)ppGpp^0^ strains, simultaneous inactivation of both regulatory pathways [(p)ppGpp^0^ Δ*codY* strain] restored virulence to near-parent-strain levels. While this finding may appear contradictory, it is in line with previous studies showing that inactivation of *codY* restores the virulence of (p)ppGpp^0^ strains of Listeria monocytogenes and Staphylococcus aureus (36, 37).

Despite the significance of the (p)ppGpp-CodY regulatory network in enterococcal CAUTI, the differences in total bacteria recovered from the infected organs observed here were not as robust as those observed in a previous investigation that depicted the essential role of the manganese transporters EfaCBA and MntH2 in CAUTI (29). Interestingly, we have shown that several phenotypes of the (p)ppGpp^0^ strain can be reverted by manganese supplementation, which is used to help the cells cope with high levels of endogenously produced reactive oxygen species (ROS) (16). Thus, it is conceivable that the attenuated virulence of the (p)ppGpp^0^ strain shown here can be traced to manganese homeostasis and thereby restored in mice fed a high-manganese diet or in animals that are unable to sequester manganese during infection (calprotectin-defective mice).

The association between (p)ppGpp and CodY, by which (p)ppGpp upregulates gene transcription via GTP depletion and the concomitant alleviation of CodY repression, has been well established (20). We previously found that the (p)ppGpp and CodY regulatory networks are also intertwined in E. faecalis by showing that inactivation of *codY* restored phenotypes of the (p)ppGpp^0^ strain, such as the inability to grow in whole blood *ex vivo* and reduced virulence in the G. mellonella invertebrate model (16). By comparing final culture pH and H_2_O_2_ production of (p)ppGpp^0^ and (p)ppGpp^0^ Δ*codY* strains, we hypothesized that deletion of *codY* restores a balanced metabolism to the (p)ppGpp^0^ strain (14, 16). The RNA-seq analysis reported here clearly supports this observation, as multiple genes coding for alternate carbon utilization pathways were strongly upregulated in the (p)ppGpp^0^ strain but not in the (p)ppGpp^0^ Δ*codY* triple mutant strain.

Our results also indicate that restoration of global metabolism in the (p)ppGpp^0^ Δ*codY* strain compared to the (p)ppGpp^0^ strain may be particularly relevant in experimental CAUTI. Specifically, we show that while the absence of either (p)ppGpp or CodY results in defects in biofilm formation *in vitro* and *in vivo*, simultaneous inactivation of the (p)ppGpp and CodY regulatory systems restores CAUTI virulence and partially restores *in vitro* biofilm formation to near-parent-strain levels (Fig. 1 and 3). Considering that the levels of the surface protein EbpA were not altered in any of the mutant strains (Fig. 3), we propose that (p)ppGpp and CodY promote biofilm formation on urinary catheters by mediating the metabolic rearrangements required for biofilm growth and survival in urine. In fact, a comparative transcriptome analysis reveals that several metabolic pathways previously identified as being relevant to enterococcal adaptation to growth in urine (38) overlap the (p)ppGpp and CodY regulons (13, 14, 20). For example, transcriptional activation of alternate carbon utilization and amino acid biosynthesis/transport genes in response to the relatively low urinary concentrations of amino acids and glucose (38–41) is shown here to be regulated by (p)ppGpp and CodY (Tables 1 and 2; see also Table S1 in the supplemental material) (13, 14, 20).

In an attempt to identify specific (p)ppGpp- and CodY-regulated processes that are important for CAUTI, we used qPCR to compare transcription levels of known virulence factors, such as the Ebp pilus and the metal transporters EfaCBA and MntH2 (27, 29), in parent and mutant strains. Although there were significant alterations in the transcription of *ebpA*, *efaC*, and *mntH2* in the (p)ppGpp and *codY* mutant strains, it was not possible to establish a firm correlation between the transcriptional expression of these virulence factors and the attenuated virulence phenotypes shown in Fig. 1. Due to the association of c-di-AMP signaling with biofilm formation (33, 34) and adaptation to osmotic stress (31, 32), two relevant traits during CAUTI, and the previous linkage with (p)ppGpp signaling (30, 35, 42), we similarly assessed gene expression of the enzymes responsible for c-di-AMP synthesis (*cdaA*) and degradation (*pde* and *gdpP*). c-di-AMP is an emerging regulatory nucleotide shown to control a number of cellular processes, including potassium homeostasis, osmotic adaptation, and biofilm formation (35). In addition, previous investigations revealed an intricate but poorly understood association between the c-di-AMP and (p)ppGpp signaling networks, in which (p)ppGpp regulates c-di-AMP levels by serving as an allosteric regulator of c-di-AMP phosphodiesterase (PDE) enzymes, while c-di-AMP stimulates (p)ppGpp synthesis via an unknown mechanism (30, 35, 42). The strong activation of *pde* and *gdpP* in the parent strain upon a shift to urine suggests that intracellular c-di-AMP levels decrease in the presence of urine. This is not surprising considering that salt concentrations in urine are generally high (average osmolarity of between 300 and 900 mosM/kg H_2_O) and that low c-di-AMP levels are associated with bacterial salt tolerance (43), whereas increased c-di-AMP levels are linked to salt hypersensitivity (44–46). Interestingly, the level of transcription of the c-di-AMP synthetase gene (*cdaA*) in urine was approximately 100-fold higher in the (p)ppGpp^0^ strain than in the parent strain, indicating that c-di-AMP homeostasis may be severely disrupted in the (p)ppGpp^0^ strain. Finally, transcription of the *pde* and *gdpP* genes was not as strongly induced (or not induced at all) in the (p)ppGpp^0^ and Δ*codY* strains. While the cellular levels of c-di-AMP in cells grown in urine remain to be determined, and the possibility of (p)ppGpp/c-di-AMP signaling cross talk in E. faecalis remains to be confirmed, the qPCR analysis suggests that E. faecalis decreases c-di-AMP pools to adjust its metabolism to the environment (i.e., high osmolality) encountered in urine.

By comparing the genes restored by *codY* inactivation in our RNA-seq analysis (Tables 1 and 2 and Table S1) to the transcriptome of E. faecalis OG1RF grown in urine (38), several other common pathways were identified, providing additional leads as to why alleviation of CodY repression restored the biofilm defect of the (p)ppGpp^0^ strain. Specifically, Vebø et al. showed that transcription of a major glycerol uptake system was induced when the OG1RF strain was grown in urine *ex vivo*, suggesting that E. faecalis utilizes glycerol as a source of energy to grow and survive in urine (38). Notably, aerobic metabolism of glycerol by the GlpO enzyme is also the main source of H_2_O_2_ generation in E. faecalis (47), and E. faecalis activates the transcription of several oxidative genes (such as *sodA*, *npr*, and *trxB*) when grown in urine (38), possibly to cope with increased ROS generation caused by aerobic glycerol metabolism. In agreement with previous microarray data (14), the current RNA-seq analysis revealed that transcription of glycerol catabolic genes, such as *glpO* and *gldA2*, was activated by 6-fold or more in the (p)ppGpp^0^ strain (Table 2 and Table S1), likely augmenting ROS production in the urinary tract. Another possibility, which is not mutually exclusive from the others, is that the strong (∼20-fold) upregulation of molybdenum metabolism genes in the (p)ppGpp^0^ strain is disadvantageous to E. faecalis when grown in the urinary tract environment. Molybdenum is a rare transition metal that functions as a cofactor of several redox-active enzymes (48–50); it should be noted that urothione, the degradation product of the molybdenum cofactor in humans, is excreted in urine (51). In contrast to other metal cofactors, molybdenum is catalytically active only when complexed with a pterin-based scaffold, forming the Moco prosthetic group (50). The Moco biosynthetic genes that are highly induced in the (p)ppGpp^0^ mutant strain code for enzymes that require GTP and the oxygen-reactive iron and copper metals (50), possibly linking molybdenum metabolism to (p)ppGpp and oxidative stress. Finally, E. faecalis appears to downregulate serine catabolism in urine (38). However, serine dehydratases were among the most highly expressed genes in the (p)ppGpp^0^ strain (∼100-fold), likely favoring pyruvate generation instead of protein synthesis. While it is unknown how serine catabolism and dysregulation of other metabolic and transport pathways affect biofilm formation and virulence in E. faecalis, alleviation of CodY repression for the most part restored the transcription of these genes to wild-type levels.

Collectively, the results presented here reveal that (p)ppGpp and CodY support the presence of E. faecalis in the catheterized murine urinary tract by controlling the metabolic arrangements necessary for the fitness of E. faecalis in this environment and, possibly, by modulating biofilm formation. Future studies using global transcriptome and metabolome analyses of (p)ppGpp-deficient and CodY-deficient strains recovered directly from CAUTI, coupled with characterization of pathways relevant to biofilm formation in urine, are necessary to fully understand how alleviation of CodY repression restores biofilm formation in the absence of (p)ppGpp. In addition, the relevance of c-di-AMP signaling to enterococcal CAUTI and the relationship between (p)ppGpp and c-di-AMP signaling pathways will warrant further investigations. In this regard, work is under way to determine the scope and targets of c-di-AMP regulation in E. faecalis. These studies should expand our mechanistic understanding of how global metabolic regulators such as CodY, (p)ppGpp, and possibly c-di-AMP mediate enterococcal pathogenesis in the urinary tract and beyond.

## MATERIALS AND METHODS

### Bacterial strains and growth conditions.

The parent E. faecalis OG1RF strain and its derivative Δ*rel*, Δ*relQ*, Δ*rel* Δ*relQ* [(p)ppGpp^0^], Δ*codY*, and (p)ppGpp^0^ Δ*codY* strains were previously described (10, 16). All strains were routinely grown in BHI broth at 37°C. For RNA sequencing (RNA-seq) analysis, cultures of E. faecalis OG1RF, (p)ppGpp^0^, and (p)ppGpp^0^ Δ*codY* strains grown overnight were diluted in a 1:100 ratio in 50 ml of chemically defined FMC medium (28) supplemented with 10 mM glucose (FMCG medium) and allowed to grow statically at 37°C to an optical density at 600 nm (OD_600_) of 0.3. Growth in pooled human urine from healthy donors (Lee Biosolutions) was monitored as described previously, with minor modifications (29). Briefly, cultures grown overnight were diluted 1:100 in phosphate-buffered saline (PBS) and inoculated at a 1:100 dilution for growth assessment in urine. Cultures were incubated aerobically at 37°C, and at selected time points, aliquots were serially diluted and plated onto tryptic soy agar (TSA) plates for CFU determination. To determine the transcriptional responses of selected genes upon transition from laboratory medium to human urine, cultures grown overnight in BHI broth were diluted 1:20 in 5 ml of fresh sterile BHI broth and allowed to grow statically at 37°C to an OD_600_ of 0.5. The bacterial cells were washed twice with PBS (pH 7.0) and pelleted down by centrifugation at 2,500 rpm for 8 min. After washing, pellets were resuspended in 7.5 ml filter-sterilized urine and incubated at 37°C for 30 min. The controls were resuspended in the same volume of fresh BHI broth and incubated at 37°C for 30 min.

### Mouse catheter implantation and infection.

The mice used in this study were 6-week-old female wild-type C57BL/6Ncr mice purchased from Charles River Laboratories. Mice were subjected to transurethral implantation and inoculated as previously described (21). Mice were anesthetized by inhalation of isoflurane and implanted with a 5-mm-long platinum-cured silicone catheter. When indicated, mice were infected immediately following catheter implantation with 50 μl of ∼2 × 10^7^ CFU of bacteria in PBS introduced into the bladder lumen by transurethral inoculation as previously described (21). To harvest the catheters and organs, mice were sacrificed at 3 days postinfection by cervical dislocation after anesthesia inhalation; the silicone catheter, bladder, and kidneys were aseptically harvested. The Washington University Animal Studies Committee approved all mouse infections and procedures as part of protocol number 20150226. All animal care was consistent with the *Guide for the Care and Use of Laboratory Animals* from the National Research Council (52).

### Biofilm formation in human urine.

For assessment of biofilm formation on fibrinogen-coated 96-well polystyrene plates (Grenier CellStar), wells were coated overnight at 4°C with 100 μg ml^−1^ human fibrinogen free of plasminogen and von Willebrand factor (Enzyme Research Laboratory). The next day, E. faecalis cultures grown overnight were diluted to an OD_600_ of 0.2 in BHI broth. The diluted cultures were centrifuged, washed three times with 1× PBS, and diluted 1:100 in urine supplemented with 20 mg ml^−1^ BSA. Bacterial cells were allowed to attach to the fibrinogen-coated plate at 37°C under static conditions as described previously (29, 53). After 24 h, microplates were washed with PBS to remove unbound bacteria, and biofilm formation was assessed by staining wells with crystal violet as previously described (53). Excess dye was removed by rinsing with sterile water, and the plates were then allowed to dry at room temperature. Biofilms were resuspended with 200 μl of 33% acetic acid, and the absorbance at 595 nm was measured on a microplate reader (Molecular Devices). Experiments were performed independently in triplicate per condition and per experiment.

### Presence of EbpA on the cell surface of (p)ppGpp- and CodY-deficient strains.

Surface expression of EbpA by E. faecalis OG1RF and derivatives was determined by an ELISA as previously described (23). Bacterial strains were grown for 18 h in urine supplemented with 20 mg ml^−1^ of BSA. Next, bacterial cells were washed (3 times) with PBS, normalized to an OD_600_ of 0.5, resuspended with 50 mM carbonate buffer (pH 9.6) containing 0.1% sodium azide, and used (100 μl) to coat Immulon 4HBX microtiter plates overnight at 4°C. The next day, plates were washed 3 times with PBS containing 0.05% Tween 20 (PBS-T) to remove unbound bacteria and blocked for 2 h with 1.5% BSA–0.1% sodium azide–PBS, followed by three washes with PBS-T. EbpA surface expression was detected using mouse anti-EbpA^Full^ antiserum, which was diluted 1:100 in dilution buffer (PBS with 0.05% Tween 20, 0.1% BSA, and 0.5% methyl-α-d-mannopyranoside) before serial dilutions were performed. A 100-μl volume was added to the plate, and the reaction mixture was incubated for 2 h. Subsequently, plates were washed with PBS-T, incubated for 1 h with horseradish peroxidase (HRP)-conjugated goat anti-rabbit antiserum (1:2,000), and washed again with PBS-T. Detection was performed using a TMB (3,3′,5,5′-tetramethylbenzidine) substrate reagent set (BD). The reaction mixtures were incubated for 5 min to allow color to develop, and the reactions were then stopped by the addition of 1.0 M sulfuric acid. The absorbance was determined at 450 nm. Titers were defined by the last dilution with an *A*_450_ of at least 0.2. As an additional control, rabbit anti-*Streptococcus* group D antiserum was used to verify that whole cells of all strains were bound to the microtiter plates at similar levels. EbpA expression titers were normalized against the bacterial titers at the same dilution.

### RNA-seq analysis.

Cells grown in FMCG medium to an OD_600_ of 0.3 were collected by centrifugation at 4,000 rpm for 20 min at 4°C and resuspended in 4 ml of a sterile RNA-stabilizing solution [3.5 M (NH_4_)_2_SO_4_, 16.6 mM sodium citrate, and 13.3 mM EDTA, adjusted to a pH of 5.2 with H_2_SO_4_]. After a 10-min incubation at room temperature, cells were centrifuged at 4,000 rpm for 30 min at 4°C, and pellets were stored at −80°C. RNA was extracted using the hot acid-phenol-chloroform method as previously described (54). Subsequently, precipitated RNA was treated once with DNase I (Ambion, Carlsbad, CA), followed by a second DNase I treatment using the DNA-free kit (Ambion) to completely remove DNA, divalent cations, and traces of the DNase I enzyme. RNA concentrations were quantified with the NanoDrop 1000 spectrophotometer (NanoDrop, Wilmington, DE), and RNA quality was assessed with the Agilent bioanalyzer (Agilent, Santa Clara, CA). RNA-seq, data processing, and statistical analysis were performed at the University of Rochester Genomics Research Center (UR-GRC) using the Illumina platform as previously described (55).

### Real-time quantitative PCR analysis.

Cultures were pelleted at 2,500 rpm for 8 min at 4°C. The bacterial pellets were resuspended in 1 ml of RNA protect and incubated for 5 min at room temperature, followed by centrifugation at 2,500 rpm for 8 min at 4°C. At that point, the bacterial pellets were kept at −80°C until ready for RNA extraction. Cells were resuspended in TE buffer (10 mM Tris Cl [pH 8], 1 mM EDTA) and 10% SDS and homogenized for three 30-s cycles, with 2 min on ice between cycles. The nucleic acids were retrieved from the total protein by phenol-chloroform (5:1) extraction. The inorganic phase was resuspended in 0.7 ml RLT buffer (Qiagen) supplemented with 1% β-mercaptoethanol, and RNA was purified using an RNeasy minikit (Qiagen), including the on-column DNase treatment recommended by the supplier. To further reduce DNA contamination, RNA samples were treated with DNase I (Ambion) at 37°C for 30 min and repurified using the RNeasy minikit (Qiagen). RNA concentrations were determined using the NanoDrop spectrophotometer. Reverse transcription and real-time PCR were carried out according to protocols described previously (54), using the primer sets indicated in Table S4 in the supplemental material.

10.1128/mSphere.00392-19.4 TABLE S4 Primers used for qPCR. Download Table S4, DOCX file, 0.01 MB. Copyright © 2019 Colomer-Winter et al. 2019 Colomer-Winter et al. This content is distributed under the terms of the Creative Commons Attribution 4.0 International license.

### Statistical analysis.

Data sets were analyzed using GraphPad Prism 6.0 software unless otherwise noted. Log_10_-transformed CFU values from urine growth curves were analyzed via two-way analysis of variance (ANOVA) followed by a comparison posttest. For CAUTI experiments, biofilm assays, and EbpA ELISAs, a two-tailed Mann-Whitney U test was performed. RNA-seq processing and the subsequent statistical analysis were performed at UR-GRC as previously described (55). qPCR data were analyzed by Student’s *t* test and ANOVA with a multiple-comparison test.

### Data availability.

Gene expression data have been deposited in the NCBI Gene Expression Omnibus (GEO) database under GEO series accession number GSE131749.
