# Supplementary material for: (p)ppGpp and CodY Promote Enterococcus faecalis Virulence in a Murine Model of Catheter-Associated Urinary Tract Infection
Source: mSphere. 2019 Jul 24;4(4):e00392-19. doi: 10.1128/mSphere.00392-19 (PMC6656871; doi:10.1128/mSphere.00392-19)
Supplement: TABLE S4 [file mSphere.00392-19-st004.docx]

**Table S4.** Primers used for qPCR.

| **Primers Sequence Application** |
| --- |

| ebpA_F | GTGGGAGCCGTCTTTGAATTG | qRT-PCR |
| --- | --- | --- |
| ebpA_R | CTCATGTCCTGCAGGTGC | qRT-PCR |
| efa_F | GTGTCGTCGGTGTGATTGTT | qRT-PCR |
| efa_R | GCAAAGAAGAAACAGCGACTAATG | qRT-PCR |
| mnth2_F | GGGAGATGTAATTAAAGGGCTGG | qRT-PCR |
| mnth2_R | GAGGCATCACTGTTGCAC | qRT-PCR |
| cdaA_F | GAACAAAAGCAGTCCAACTATTAAAAGG | qRT-PCR |
| cdaA_R | GCCTGAATAATACGCTCATCTTCTTG | qRT-PCR |
| pde_F | TGAACCCTATGGAGATTTAGTTTGG | qRT-PCR |
| pde_R | TGGTCTAACTCACGATTTAAGTCTGC | qRT-PCR |
| gdpP_F | GCCAAGTTACGTATTTAGAATTAGCAG | qRT-PCR |
| gdpP_R | AGACACAATGCCAATAGCCGTTTG | qRT-PCR |
